# Proper time regularization and the QCD chiral phase transition

**DOI:** 10.1038/srep45937

**Published:** 2017-04-12

**Authors:** Zhu-Fang Cui, Jin-Li Zhang, Hong-Shi Zong

**Affiliations:** 1 Department of Physics, Nanjing University, Nanjing, 210093, China; 2 State Key Laboratory of Theoretical Physics, Institute of Theoretical Physics, CAS, Beijing, 100190, China; 3 Joint Center for Particle, Nuclear Physics and Cosmology, Nanjing 210093, China

## Abstract

We study the QCD chiral phase transition at finite temperature and finite quark chemical potential within the two flavor Nambu–Jona-Lasinio (NJL) model, where a generalization of the proper-time regularization scheme is motivated and implemented. We find that in the chiral limit the whole transition line in the phase diagram is of second order, whereas for finite quark masses a crossover is observed. Moreover, if we take into account the influence of quark condensate to the coupling strength (which also provides a possible way of how the effective coupling varies with temperature and quark chemical potential), it is found that a CEP may appear. These findings differ substantially from other NJL results which use alternative regularization schemes, some explanation and discussion are given at the end. This indicates that the regularization scheme can have a dramatic impact on the study of the QCD phase transition within the NJL model.

Strongly interacting matter – which is described by Quantum ChromoDynamics (QCD) – is believed to have a rich phase structure. In the low temperature (*T*) and low quark chemical potential (*μ*) region the system is usually named as Nambu (or Nambu-Goldstone) phase, where dynamical chiral symmetry breaking (DCSB) and color confinement of quarks and gluons are the key emergent phenomena. Correspondingly, the high *T* and/or high *μ* phase is often called Wigner (or Wigner-Weyl) phase, where the chiral symmetry is (partially) restored, and is then thought to be related to the theoretically predicted quark-gluon plasma (QGP) state. The discussions of QGP, in which (partially) deconfined colored quarks and gluons may appear, is under active experimental study at relativistic heavy-ion colliders such as RHIC and LHC, which create fireballs of very high temperature (∼200 MeV). Furthermore, studies at high *T* and low *μ* are relevant to the evolution of the early Universe, while the low *T* and high *μ* regime is manifest in the central core of compact stars.

The non-perturbative nature of low energy QCD makes it difficult, or even impossible, to study many aspects of the QCD phase diagram from first principles. For example, for the QCD phase diagram beyond the chiral limit (that is for finite quark masses), a popular scenario is a crossover at finite *T* and low *μ*, which turns into first order for larger *μ* at a possible critical end point (CEP). The search for such a CEP is one of the main goals in the high energy physics experiments, such as the beam energy scan (BES) program^1^,^2^,^3^,^4^,^5^. However, there is no agreement on the numerical value for the CEP^6^ and no clear agreement on the physical picture.

Early on it was thought that the quark hadron transition is of first order, even at low *μ*, which led to an important proposal by Witten regarding the possible formation of quark nuggets^7^. Subsequently, lattice QCD has shown that this transition is most likely a crossover, at least for the low *μ* region^8^,^9^,^10^. Nevertheless, at present most lattice calculations are performed with non-chiral fermions which only recover chiral symmetry when the lattice spacing is taken to zero, and are not suitable for the finite *μ*/*T* region because of the fermion sign problem. Moreover, in refs 11,12,13 it is argued that there may be two CEPs and the authors of ref. 14 state that there is no CEP but instead a Lifshitz point. In ref. 15 it is argued that there is no CEP since the phase transition is a crossover in the whole phase diagram and the authors of refs 16,17,18 have demonstrated that either a crossover, or a first (or second) order phase transition is possible, depending on the model setup and/or choice of the parameters. How the regularization scheme and parameter choices affect the phase diagram is studied in refs 19, 20, 21, 22, 23. Refs 24,25 show that for a sufficiently asymmetric system the CEP is not present. The authors of ref. 26 have found that at low volume the CEP is pushed towards higher *μ* and lower *T* domain, then at *R* = 2 fm the CEP vanishes and the whole phase diagram becomes a crossover. Studies in ref. 27 also find no evidence for a CEP. Some recent debates can be found in refs 28,29,30.

The non-Abelian nature of QCD makes it difficult to have a thorough understanding of DCSB and confinement, especially in the infrared domain where the coupling strength becomes large. In this regime one must often resort to various effective models to study QCD, which inevitably introduces model dependence. Prominent examples are the Nambu–Jona-Lasinio (NJL) model^19^,^20^,^22^ and the Dyson-Schwinger equations (DSEs)^31^,^32^,^33^,^34^, which are powerful and popular tools for the study of hadron physics and phase transitions of strongly interacting matter. The NJL model, which describes interactions between dressed quarks,has been shown to describe most chiral properties of the system. To cure the ultraviolet (UV) divergence of this model, the covariant proper-time regularization (PTR) is adopted in this work.

## Model and Methods

The phase structure of strongly interacting matter has been actively investigated using various effective models. In this section we give a basic introduction to the NJL model, which is widely regarded as a faithful phenomenological model of QCD that works rather well in describing the quark dynamics up to intermediate energies. In particular, it exhibits the feature of DCSB, which is responsible for the dynamical mass generation from bare quarks. In this effective model, the Lagrangian is constructed in such a way that the basic symmetries of QCD, which are observed in nature, are part and parcel of it, while all interaction terms are simplified to be four-body interactions. Usually, the following Lagrangian density is introduced, which is proved to work well in the region of intermediate length between the asymptotic freedom and confinement regions (throughout this work we will always work in Euclidean space, and take the number of flavors *N*_f_ = 2, the number of colors *N*_c_ = 3),


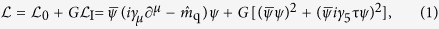


where *ψ* = (*ψ*_u_, *ψ*_d_)^T^ is the quark field, and the mass matrix is 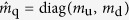. We will work in the limit of exact isospin symmetry, namely, *m*_u_ = *m*_d_ ≡ *m*. Here a local, chirally symmetric scalar-pseudoscalar four-point interaction of the quark fields is introduced with an effective coupling strength *G* (some discussions for a varying coupling strength can be found in refs 35,36,37).

With the mean field approximation of Eq. (1), the effective quark mass *M* can be determined via the self-consistent gap equation,


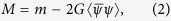


where the two-quark condensate is defined as


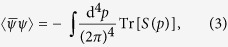


in which *S(p*) is the dressed quark propagator and the trace is to be taken in color, flavor, and Dirac spaces. Lorentz covariance implies that for *T* = *μ* = 0 the general form of *S(p*) is


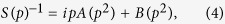


where *A(p*^2^) and *B(p*^2^) are scalar functions. It is easy to show from Eq. (2) that the solution is *A(p*^2^) = 1 and *B(p*^2^) = *M*.

The successes of NJL model indicate that a simple model can often capture the essential physics of QCD, nevertheless, it has two main shortcomings: it is neither confining nor renormalizable (a result of the contact interaction). For the former, usually this model is expected to work well only in the regions where confinement effects may not be essential, and some treatments can be used to mimic confinement^38^,^39^,^40^,^41^,^42^,^43^,^44^. For the latter one, people often introduce some kind of momentum scale that the interactions have as a cutoff, so that all the possible UV divergences can be avoided. This is an acceptable procedure only if we assume that the cutoff is much larger than all the relevant momenta. One choice of such regularization schemes is the non-covariant but physically intuitive three-momentum cutoff, while there are also some covariant ways (so that they have the attractive feature of being Lorentz invariant) like the four-momentum cutoff, the Pauli-Villars method, and the proper time regularization that this work uses. Detailed discussions and comparisons of these regularization schemes can be found in refs 19,22. The key equation of the proper time regularization is


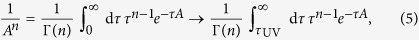


where *A* > 0 is assumed, *τ*_U*V*_ = 1/Λ_U*V*_^2^, Λ_U*V*_ is the _U*V*_ cutoff, and the (Euler-) Gamma function is defined as


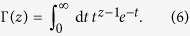


Here it should be noted that, the infrared cutoff (Λ_IR_), which is introduced to ensure confinement in refs 38, 40, 41, 42, 43, is assumed to be zero in our study, since the confinement effects is not crucial in our calculations related to quark degrees of freedom. In order to avoid unphysical thresholds for the decay of hadrons (nucleons) into quarks, the low value of the constituent quark mass, for the case *μ* = *T* = 0, would demand a nonzero infrared cutoff in the hadronic phase. Using Eq. (5), the four-momentum *p* = (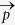, *p*_4_) can be treated in a covariant way, and the related integrals will become finite, for example


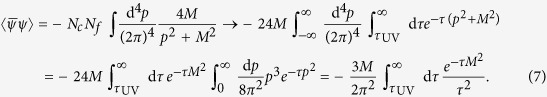


Then the gap equation (2) becomes


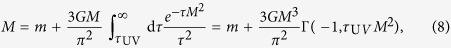


here the incomplete Gamma function is defined as


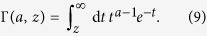


This model has three parameters: the “bare” quark mass *m*, the coupling strength *G*, and the UV cutoff Λ_UV_. Usually, people fix them by the requirement of reproducing the known chiral physics in the vacuum, such as the two-quark condensate derived from QCD sum rules or lattice QCD, the pion decay constant *f*_*π*_, or the Gell-Mann–Oakes–Renner (GMOR) relation, 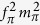 = −*m*〈
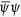
〉, etc., details can be found in refs 19,22. In this work we use the second case in Table 1. of ref. 43, that *m* = 5.0 MeV, *G* = 3.26 × 10^−6^ MeV^−2^, and Λ_UV_ = 1080 MeV, which give *f*_*π*_ = 92 MeV (with *m*_*π*_ = 138 MeV), *M* = 216 MeV, and 〈
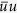
〉^1/3^ = −253 MeV. Further calculations show that for different parameter sets there are only quantitative differences in our study, as we will discuss in Sec. Discussion.

According to finite temperature quantum field theory, the extension of vacuum study to the cases with finite *T* and *μ* is systematically accomplished by transcription of the four-momentum via 
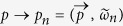
, where 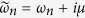, *ω*_*n*_ = (2*n* + 1)*πT* with *n*∈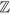 the fermion Matsubara frequencies. In this case, if one still wants to treat *p*_*n*_ in a covariant way, for the case of finite *T* and *μ* = 0 the calculation is straightforward^42^,^43^, while for *μ* ≠ 0 it is quite complicated, since now the denominator *p*^2^ + *M*^2^ in Eq. (7) becomes *w*_*n*_^2^ + 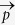
^2^ + *M*^2^ − *μ*^2^ + 2*iμw*_*n*_. Then in addition to treating the imaginary part 2*iμw*_*n*_ we also have to determine if *w*_*n*_^2^ + 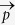
^2^ + *M*^2^ − *μ*^2^ is positive or otherwise for each *n* and *μ*^22^. Moreover, at the cases with *T* ≠ 0 and/or *μ* ≠ 0, since the previous *O*(4) symmetry is broken down to *O*(3), the system is no longer covariant, so we argue that in principle the fourth component of the momentum should be integrated out first, as in the three-momentum noncovariant cutoff regularization scheme, otherwise the UV cutoff should vary as some proper function of *T* and *μ*, which is more complicated and even impossible at present. Using such treatment, it is easy to verify that the two-quark condensate at *T* = *μ* = 0 and vacuum properties of pion are still the same as above (because the integral for the momentum is always from 0 to ∞). The two-quark condensate for *T* ≠ 0 and *μ* ≠ 0 then takes the form


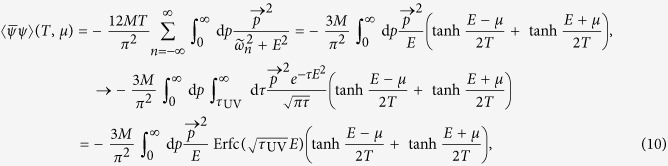


here *E* = (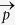
^2^ + *M*^2^)^1/2^, Erfc(*x*) = 1 − Erf(*x*) gives the complementary error function. Note, the error function Erf(*x*) is the integral of the Gaussian distribution,


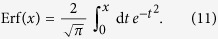


It is easy to verify that in the *T* → 0 and *μ* → 0 limit, Eq. (10) becomes to Eq. (7). Moreover, in this regularization treatment the summation of all the Matsubara frequencies are carried out even for the *μ* ≠ 0 case, which then avoids the complication in the four-momentum cutoff as well as the proper time regularization in ref. 22, and more self-consist with the treatment of the three-momentum part.

We can also discuss the case with *T* = 0 and *μ* ≠ 0, where the two quark condensate becomes a piecewise function


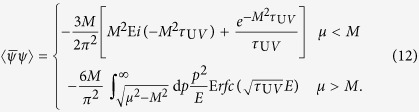


with Ei(*x*) = −∫_−*x*_^∞^(*e*^−*t*^)/(*t*)d*t*, where the principal value of the integral is taken. We can see clearly from Eq. (12) that *M* is independent of *μ* when *μ* < *M*, which means the vacuum is unchanged for lower *μ*, which is in consistent with the model independent result of ref. 45.

## Results

**Figure 1:**
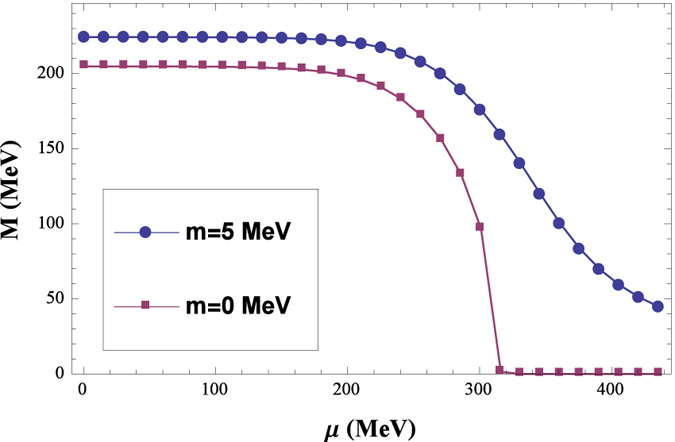
Dependence of the effective quark masses *M* as functions of *μ* for *T* = 30 MeV.

We now solve Eqs (2) and (10) by numerical iteration, and it should be stressed here that, since we work with quark degrees of freedom, and no confinement effects are included, all our results and discussion are about the chiral phase transition of QCD. As an example, the result of *T* = 30 MeV is shown in Fig. 1, which indicates a second order phase transition in the chiral limit case and a crossover for finite current quark mass. It can be seen that for both cases, the effective quark masses decrease monotonously as *μ* increases, which means that the dressing effects of quarks become increasingly weak at higher *μ*. Furthermore, both of the effective quark masses decrease slowly at low *μ* first (almost a constant for *μ* < 200 MeV, which means the vacuum for *T* =30 MeV becomes nontrivial only when *μ* >200 MeV), and begin to decrease rapidly for some ranges of *μ*, then become flatter for larger *μ*.

From Fig. 1 we can also see clearly that, in the chiral limit, the critical chemical potential *μ*_*c*_ (for *T* = 30 MeV) is about 315 MeV, where the second order phase transition happens, while for the case with finite quark mass the critical chemical potential is hard to determine, since in this case there is no real phase transition and the critical behavior become ambiguous. To study the properties of the crossover region various QCD susceptibilities are usually employed, which are the linear responses of the QCD condensate to various external fields^46^. For example, the scalar susceptibility (or usually called chiral susceptibility, since it is often used to study the QCD chiral phase transition) is the linear response of the two-quark condensate to the scalar external field, defined as


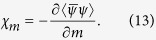


**Figure 2:**
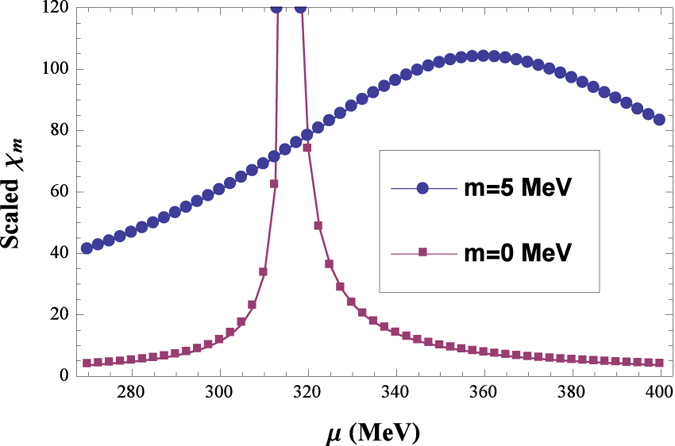
Dependence of the scalar susceptibilities *χ*_*m*_ as functions of *μ* for *T* = 30 MeV, here the result of *m* =5 MeV is multiplied by 20.

The results for the scalar susceptibilities with *T* = 30 MeV are shown in Fig. 2, to be clear, the result for *m* = 5 MeV is multiplied by 20. For the chiral limit case, we can see that there is a divergence around *μ* = 315 MeV, which indicates a second order phase transition, while beyond the chiral limit, there is only a finite peak around *μ* = 360 MeV for a continuum line, which is just the typical characteristic of a crossover.

**Figure 3:**
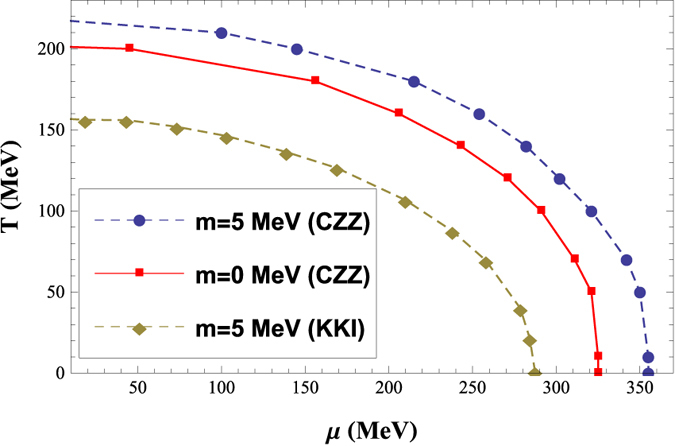
Chiral phase diagram based on our model study. As a comparison, the corresponding result of ref. 22 is also shown as the KKI line.

Based on the results of Figs. 1 and 2, we can calculate the whole chiral phase diagram, as show in Fig. 3. For the chiral limit case, the results show that the whole phase transition line is second order, and the critical *μ* for different *T* we choose are just the second order phase transition points; while beyond the chiral limit the whole phase diagram is a crossover, we choose the corresponding *μ* of the peak of every scalar susceptibility for each *T*, which are just the “pseudo-critical” chemical potentials. As a comparison the corresponding result of ref. 22 is also shown as KKI line. We can see that for this parameter set our result is qualitatively quite similar, just pushed to higher *T* and *μ*. It is interesting to note that our result for *T* = 0 seems to be better, since a model independent argument is that the vacuum is trivial under some critical *μ*, and the phase transition should take place after roughly *μ* > *m*_*N*_/3 ≈ 310 MeV for the lightest quarks (*u* and *d*)^45^. On the other hand, the *μ* = 0 result of ref. 22 is more close to the lattice QCD calculation^47^. We stress that our quantitative results of Fig. 3 may vary for different parameter sets, but the qualitative behaviors are the same, so that the results we show here are representative for our model setup (more discussion can be found in the Discussion Section). At the same time, a CEP may appear for other parameter choice in ref. 22.

**Figure 4:**
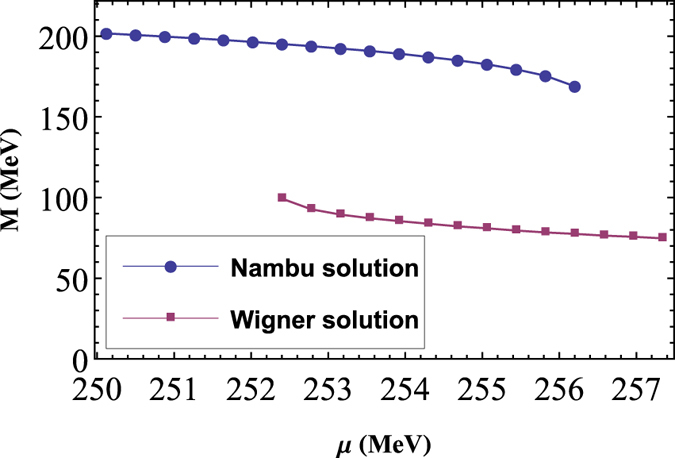
Multi-solution of the quark gap equation Eq. (2) with *G*1 = 0.84 *G* and *T* = 0, for the *m* =5 MeV case. It can be seen clearly that, Nambu and Wigner solutions could coexist between about 252.5 MeV to 256.3 MeV.

**Figure 5:**
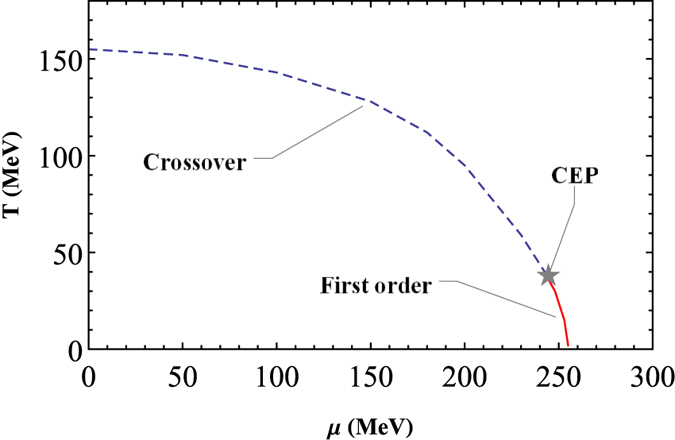
Chiral phase diagram with *G*1 = 0.84 *G*, for the *m* = 5 MeV case.

Following the idea of refs 35,36,37, here we discuss the case that the effective coupling strength *G* is replaced by *G(T, μ*) = *G*1 + *G*2〈
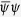
〉(*T, μ*), where *G*1 and *G*2 are new constants. As a comparison, we set *G*(0, 0) = *G*, and when *T* and/or *μ* are nonzero, the *G*2〈
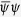
〉(*T, μ*) part can then characterize the influence of quark condensates to the coupling strength of quark interaction. Moreover, this also provides a possible way of how the effective coupling vary with *T* and *μ*, and it is easy to know that *G(T, μ*) would become smaller for higher *T* and/or *μ*, which qualitatively agree with the renormalization group arguments^48^. It is interesting that, for *G*1 less than about 0.85*G*, the phase transition of the low *T* and high *μ* region would become first order, which indicates that a CEP would appear. As an example, we show the case of *G*1 = 0.84*G* and *T* = 0 in Fig. 4 for the *m* = 5 MeV case, where obvious multi-solution of the gap equation is shown, and the first order phase transition point would take place at the middle of the multi-solution region, where the corresponding thermodynamic potentials and chiral susceptibilities of these two solutions would equal each other. Here, the one with larger effective mass is often named as “Nambu-Goldstone” solution or “Nambu” solution, which describes the phase where chiral symmetry is broken, and then should disappear when µ is larger than some critical value; while the one with smaller effective mass is called “Wigner-Weyl” solution or “Wigner” solution, which corresponds to the phase that chiral symmetry is (partially) restored, so only should appear after some other critical value of µ, and then could exist to infinity. In other words, besides smaller than Nambu solution, Wigner solution should be 0 in the chiral limit, while Nambu solution does not. Based on this, we can draw a possible chiral phase diagram for the *m* =5 MeV case, as shown in Fig. 5, where *T*_*c*_ for *μ* = 0 is around 155 MeV, close to the lattice QCD calculation^47^, and CEP is located at (*T, μ*) =(38 MeV, 245 MeV). Then, if *G*1 is smaller than some critical value, the whole phase diagram would become first order, as discussed in refs 35,36.

As we have discussed, since lattice QCD, one of the most important non-perturbative frameworks at present, has the thorny “sign problem”, any information from the compact stars is quite useful, since their central cores are supposed to reach the baryon density relevant to the hadron-quark phase transition. Therefore compact stars, especially neutron stars, represent a natural and unique environment for investigating extremely dense strongly interacting matter at relatively low temperatures. On the other hand, the physics of neutron stars is also one of the central areas of research in nuclear astrophysics. In literature of neutron star studies, the crossover behavior in the hadron quark transition are also discussed, see for example, refs 49, 50, 51, 52, 53, 54, 55, 56, 57, 58, which can explain some of the neutron star properties well, like the mass-radii relations.

## Discussion

In this paper, we have generalized the proper time regularization to the case with finite temperature and finite quark chemical potential within the two flavor NJL model, also drawn a possible QCD chiral phase diagram for both beyond and in the chiral limit cases based on our calculation. The case that the effective quark coupling strength is related to quark condensates is also discussed, which also provides a possible way of how the effective coupling vary with *T* and *μ*, and qualitatively agrees with the renormalization group arguments. It is found that for some cases a CEP would appear. As we discussed above, owing to the non-perturbative nature of QCD, the phase diagram at finite temperature and baryon density is still largely unknown today. Lattice QCD, the best first principle calculation at present, suffers from a severe sign problem when chemical potential for baryon number is non-vanishing. In order to reach a comprehensive understanding of QCD and our nature, methods are required which may at least potentially solve this problem.

**Figure 6:**
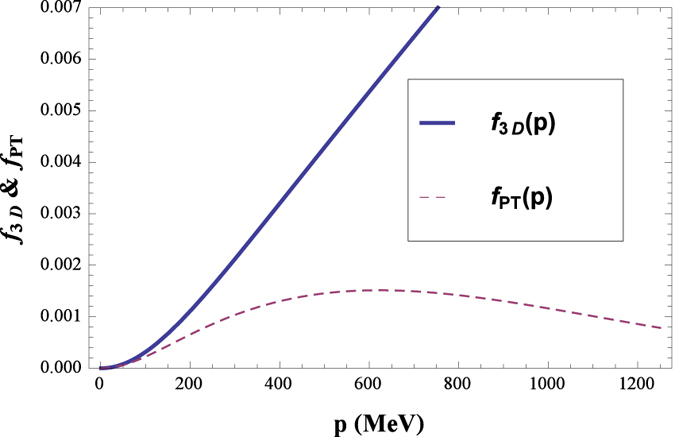
Sketch comparison of the integrands of the three-momentum cutoff regularization (*f*_3*D*_) and our proper time regularization (*f*_*PT*_), the qualitative behavior not sensitive for the precise values of *T* and *μ*.

Actually, the main difference of our regularization procedure and the three-momentum cutoff regularization comes from Eq. (10), where the latter does not have the Erfc function, and an upper limit for the momentum *p* is imposed. Here we define the integrands as


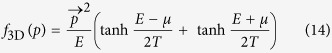



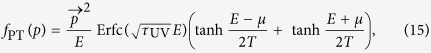


a diagrammatic sketch of the difference between *f*_3*D*_(*p*) and *f*_P*T*_(*p*) is depicted in Fig. 6, while this comparison is not qualitatively sensitive to the precise values of *T* and *μ*. We can see that for large *p, f*_3*D*_(*p*) ∝ *p*, as can be understood easily from its definition (Equation (14)), hence a momentum cutoff is unavoidable; while for *f*_P*T*_(*p*), the Erfc function make it tends to 0 for large *p*, then the integration of *p* does not need any cutoff. Moreover, it can be proven numerically that if the effect of the Erfc function is decreased (at the same time, a proper momentum cutoff should appear), a CEP will appear for some parameter choice, as in the three-momentum cutoff regularization. Our results show that, the calculation of the QCD phase diagram is quite model setup dependent, at least within the NJL model, where different regularization scheme may lead to qualitatively different results (Besides, the relation between the NJL model and QCD itself remains somewhat obscure, accordingly the qualitative results are often more important than the quantitative ones). As we all know, in a renormalizable quantum field theory, the regularization procedures should not affect any physical outcome; however, if the model being discussed is not renormalizable, corresponding regularization procedure and cutoff(s) will inevitably affect outcomes. In this sense, one has to choose a proper model as well as regularization procedure for some specific issue.

## Additional Information

**How to cite this article**: Cui, Z.-F. *et al*. Proper time regularization and the QCD chiral phase transition. *Sci. Rep.* **7**, 45937; doi: 10.1038/srep45937 (2017).

**Publisher's note:** Springer Nature remains neutral with regard to jurisdictional claims in published maps and institutional affiliations.
